# Characterization of m^6^A RNA Methylation Regulators Predicts Survival and Immunotherapy in Lung Adenocarcinoma

**DOI:** 10.3389/fimmu.2021.782551

**Published:** 2021-12-17

**Authors:** Minggao Zhu, Yachao Cui, Qi Mo, Junwei Zhang, Ting Zhao, Yujie Xu, Zhenpeng Wu, Donglin Sun, Xiaoren Zhang, Yingchang Li, Qiang You

**Affiliations:** ^1^ Affiliated Cancer Hospital & Institute, Guangzhou Medical University, Guangzhou, China; ^2^ Key Laboratory of Cell Homeostasis and Cancer Research of Guangdong Higher Education Institutes, Guangzhou Medical University, Guangzhou, China; ^3^ Center for Cancer and Immunology Research, State Key Laboratory of Respiratory Disease, Guangzhou, China

**Keywords:** m^6^A, lung adenocarcinoma, tumor microenvironment, tumor-infiltrating immune cells, tumor mutation burden, radiotherapy, immunotherapy

## Abstract

N^6^-methyladenosine (m^6^A) RNA modification is a reversible mechanism that regulates eukaryotic gene expression. Growing evidence has demonstrated an association between m^6^A modification and tumorigenesis and response to immunotherapy. However, the overall influence of m^6^A regulators on the tumor microenvironment and their effect on the response to immunotherapy in lung adenocarcinoma remains to be explored. Here, we comprehensively analyzed the m^6^A modification patterns of 936 lung adenocarcinoma samples based on 24 m^6^A regulators. First, we described the features of genetic variation in these m^6^A regulators. Many m^6^A regulators were aberrantly expressed in tumors and negatively correlated with most tumor-infiltrating immune cell types. Furthermore, we identified three m^6^A modification patterns using a consensus clustering method. m^6^A cluster B was preferentially associated with a favorable prognosis and enriched in metabolism-associated pathways. In contrast, m^6^A cluster A was associated with the worst prognosis and was enriched in the process of DNA repair. m^6^A cluster C was characterized by activation of the immune system and a higher stromal cell score. Surprisingly, patients who received radiotherapy had a better prognosis than patients without radiotherapy only in the m^6^A cluster C group. Subsequently, we constructed an m^6^A score model that qualified the m^6^A modification level of individual samples by using principal component analysis algorithms. Patients with high m^6^A score were characterized by enhanced immune cell infiltration and prolonged survival time and were associated with lower tumor mutation burden and PD-1/CTLA4 expression. The combination of the m^6^A score and tumor mutation burden could accurately predict the prognosis of patients with lung adenocarcinoma. Furthermore, patients with high m^6^A score exhibited greater prognostic benefits from radiotherapy and immunotherapy. This study demonstrates that m^6^A modification is significantly associated with tumor microenvironment diversity and prognosis. A comprehensive evaluation of m^6^A modification patterns in single tumors will expand our understanding of the tumor immune landscape. In addition, our m^6^A score model demonstrated that the level of immune cell infiltration plays a significant role in cancer immunotherapy and provides a basis to increase the efficiency of current immune therapies and promote the clinical success of immunotherapy.

## Introduction

Lung cancer remains one of the most difficult-to-treat cancers, and its morbidity and mortality are rising rapidly (1). Lung adenocarcinoma (LUAD) accounts for approximately 40% of all lung cancers (2). Driver genes in LUAD include *RTKs* (aberrantly expressed), *EGFR/KRAS* (mutations), *ALK* (rearrangement), and others (3–6). Despite recent advances in surgery, radiotherapy, chemotherapy, targeted therapy, and immunotherapy, the prognosis of patients with LUAD is still unsatisfactory (7). LUAD is a complicated disease with complex pathogenesis and high heterogeneity (8). Therefore, having a good understanding of the molecular mechanisms underlying LUAD is necessary for the selection of optimal therapeutic strategies.

N^6^-methyladenosine (m^6^A) RNA modification has recently been identified as a regulatory mechanism for controlling eukaryotic gene expression (9). As a dynamic reversible epigenetic modification, m^6^A modification exists in mRNAs, microRNAs, circular RNAs, and long noncoding RNAs, accounting for 80% of all RNA methylation modifications in eukaryotic cells (10). m^6^A modification is mediated by three subtypes of regulatory proteins: methyltransferases (writers), binding proteins (readers), and demethylases (erasers) (11). The modification is mainly regulated by the following components: writers, which catalyze m^6^A methylation, such as methyltransferase-like 3/14/16 (*METTL3/14/16*) (12–14), zinc finger CCCH domain-containing protein 13 (*ZC3H13*) (15), ELAV-like RNA-binding protein 1 (*ELAVL1*) (16), Cbl proto-oncogene-like 1 (*CBLL1*) (17), RNA-binding motif protein 15/15B (*RBM15/15B*) (17, 18), WT1-associated protein (*WTAP*) (19), and VIR-like m^6^A methyltransferase associated (*KIAA1429*) (20). Erasers are proteins involved in maintaining the balance of the m^6^A content in the transcriptome and include fat mass and obesity-associated protein (*FTO*) (21) and AlkB homolog H5 (*ALKBH5*) (22). Readers are proteins that recognize the m^6^A consensus motif (DRACH) and promote stimulatory and inhibitory effects on translation dynamics, such as YTH domain family 1/2/3 (*YTHDF1/2/3*) (23, 24), YTH domain containing 1/2 (*YTHDC1/2*) (25, 26), IGF2 mRNA-binding proteins 1/2/3 (*IGF2BP1/2/3*) (27, 28), Fragile X mental retardation 1 (*FMR1*) (29), leucine-rich pentatricopeptide repeat containing (*LRPPRC*) (29), heterogeneous nuclear ribonucleoprotein C (*HNRNPC*) (30), and heterogeneous nuclear ribonucleoprotein A2/B1 (*HNRNPA2B1*) (31). m^6^A regulators posttranscriptionally modify RNA molecules and are associated with many biological processes, including carcinogenesis, immune response, cell differentiation, neurodevelopment, and stress responses (9). In addition, m^6^A mRNA modification may play a significant role in the occurrence and development of human cancers (32), such as lung cancer, hepatic cell carcinoma, and glioblastoma. *METTL3* directly promotes *YAP* translation and increases its activity, which induces resistance to nonsmall cell lung cancer drugs and metastasis (33). Moreover, the upregulation of *WTAP* contributes to hepatocellular carcinoma tumorigenesis by repressing *ETS1* expression (34). Furthermore, the m^6^A demethylase *ALKBH5* maintains the tumorigenicity of stem-like cells by supporting cell proliferation and *FOXM1* expression in glioblastoma (35). However, the relationship between m^6^A modulators and tumors, especially immunotherapy, remains unclear. Therefore, further elucidation of m^6^A regulatory factors could provide an attractive perspective for cancer therapy (36).

Immune checkpoint therapy has shown unprecedented efficacy for various malignancies by boosting the immune system to fight cancer (37). Immune checkpoints refer to a plethora of inhibitory or stimulatory molecules that maintain self-tolerance, prevent autoimmunity, and control the duration and extent of immune responses, which are hijacked by cancer cells to evade immune eradication (38–40). Immune checkpoint inhibitors (ICIs) target these checkpoints and show remarkable clinical efficacy in a broad spectrum of tumors. Unfortunately, only a considerable proportion of patients receive clinical benefits from ICIs (41). In recent years, many studies have demonstrated the correlation between tumor-infiltrating immune cells and m^6^A modification patterns, which cannot be explained by RNA degradation mechanisms. Wang et al. reported that the inhibition of m^6^A modification could enhance the response to anti-PD-1 therapy in pMMR-MSI-L CRC and melanoma (42). *ALKBH5* gene expression and mutation status are correlated with response to immunotherapy in melanoma patients, demonstrating that m^6^A erasers influence the therapeutic effects of immunotherapy (43). However, the overall impact of all m^6^A regulators on the immune microenvironment and their effect on the response to immunotherapy are still unclear.

In this study, we analyzed genomic information from 936 patients with LUAD to determine their methylation modification patterns. In addition, we constructed an m^6^A score model to quantify the m^6^A modification patterns of individual tumors and predict the clinical response to ICI treatment. Our findings clarify the important role of m^6^A methylation in LUAD and provide clues for improving the efficiency of current immune therapies, which will contribute to the selection of an effective personalized immunotherapy strategy.

## Materials and Methods

### Data Source and Processing

The expression matrices and corresponding clinical characteristics of LUAD samples were obtained from the Gene Expression Omnibus (GEO) and The Cancer Genome Atlas (TCGA) databases. We excluded patients without survival, information, or incomplete clinicopathological characteristics from further assessment. A total of 936 patients were enrolled, including the cohorts GSE68465 (*N* = 438) and TCGA-LUAD (*N* = 498). The four validation databases were downloaded from the GEO database, including GSE11969, GSE13213, GSE37745, and GSE50081. The expression matrix data of the TCGA-LUAD cohort (FPKM format) were downloaded from the Genomic Data Commons platform, and FPKM units were converted to transcripts per kilobase million (TPM) units. The “Normalized Between Arrays” function of the R package “Limma” was performed for data standardization. Genome mutation data of TCGA-LUAD (including somatic mutation and copy number variation (CNV)) were downloaded from the UCSC Xena platform. Based on previous studies, we collected 24 m^6^A regulators, including 10 writers (*CBLL1*, *ELAVL1*, *METTL3*, *METTL14*, *METTL16*, *KIAA1429*, *RBM15*, *RBM15B*, *WTA*, and *ZC3H13*), two erasers (*ALKBH5* and *FTO)*, and 12 readers (*YTHDC1*, *YTHDC2*, *YTHDF1*, *YTHDF2*, *YTHDF3*, *FMR1*, *HNRNPA2B1*, *HNRNPC*, *IGF2BP1*, *IGF2BP2*, *IGF2BP3*, *LRPPRC*). Clinicopathological information and clinical immunotherapy scores (IPS) were obtained from the TCIA database.

### m^6^A Modification Pattern

Based on mRNA expression levels, 19 m^6^A regulators were extracted from the TCGA-LUAD and GSE68465 cohorts, and the samples were divided into diverse subtypes based on transcriptome data with the R package “Consensus Cluster Plus.” A thousand repetitions were performed to guarantee the stability of the classification (44).

### Pathway Enrichment Analysis

To investigate the difference between m^6^A modification patterns in the biological process, we explored the variation in signaling pathways between each of the two subtypes of m^6^A regulators by using “Gene set variation analysis (GSVA)” R packages (45). We downloaded the gene set file “c2.cp.kegg.v7.4.symbols” from the MSigDB database for the GSVA analysis. An adjusted *p*-value of less than 0.05 was considered statistically significant.

### Analysis of Immune Cell Infiltration

To estimate the relative abundance of 23 immune cell types in the tumor microenvironment of LUAD, single-sample gene set enrichment analysis (ssGSEA) was used to calculate the enrichment scores and represent the relative abundance of each tumor-infiltrating immune cell type in each sample. The set of genes used to label each tumor-infiltrating immune cell type was obtained from the study by Charoentong (46).

### Identification of Differentially Expressed Genes Among Subtypes of m^6^A Regulators

The patients were divided into three subtypes according to the expression level of 19 m^6^A regulators. We identified differentially expressed genes (DEGs) among different subtypes using the empirical Bayesian method in the R package “Limma,” and selected *p*-values less than 0.001 as DEG candidates for further analysis (47).

### Construction of the m^6^A Score Model

To quantify the level of m^6^A modification in a single tumor, we established an m^6^A score model by performing principal component analysis (PCA). The procedure for the established m^6^A score model was as follows: first, overlapping DEGs were identified from different m^6^A clusters, and significant prognosis-related genes were identified by univariate Cox regression analysis; second, PCA was performed on the gene expression profile, and the principal components 1 and 2 were extracted as feature scores. This method minimized the deprivation of information contained in the original index and reduced the indicators to be analyzed, thereby allowing a comprehensive analysis of the collected data; lastly, the m^6^A score was defined by performing a formula similar to that used in previous studies (48, 49). m^6^A score = ∑ (PC1*i* + PC2*i*), where *i* is the expression of m^6^A phenotype-related genes.

### Statistical Analysis

All statistical analyses were performed with R software (version 4.0.3). The R package “Limma” was used for differential gene expression analysis. Spearman’s correlation analysis was used to calculate the correlation coefficients between the levels of different tumor-infiltrating immune cell types and the expression of m^6^A regulators. Kruskal-Wallis test and one-way analysis of variance were used to compare differences between more than two groups. The Kaplan-Meier (KM) method was used to draw the survival curve (5-year survival rate), and univariate Cox regression analysis was used to calculate the hazard ratios (HRs) for m^6^A regulators and m^6^A phenotype-related genes. LUAD samples were divided into high and low m^6^A score subgroups using the “surv-cutpoint” function in the R package “survival.”

## Results

### Genetic m^6^A Regulator Variation in LUAD

**Figure 1:**
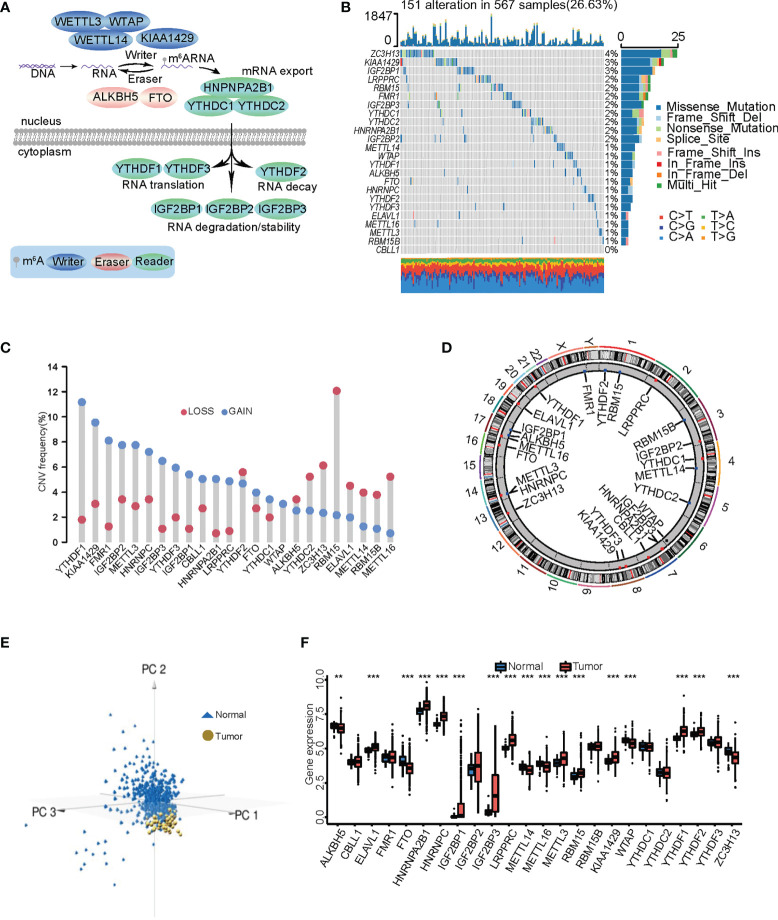
Gene mutation profile and expression of m^6^A regulators in lung adenocarcinoma (LUAD). **(A)** The dynamic and reversible modification process of m^6^A RNA methylation mediated by 24 m^6^A regulators and their major biological functions. **(B)** The mutation frequency of 24 m^6^A regulators in 567 patients with LUAD. Each column represents each individual patient. The upper bar plot represents TMB. The number on the right represents the mutation frequency in each regulator. The bar graph on the right shows the proportion of each variant type. The stacked bar chart below shows the conversion of each sample. **(C)** Histogram reflecting the CNV of the m^6^A regulators. The height of the bar indicates the frequency of variation. Gain, blue; loss, red. **(D)** The specific location of the CNVs in m^6^A regulators on 23 chromosomes. **(E)** Principal component analysis of 24 m^6^A regulators adapted to distinguishing tumors from normal samples. Tumor was marked with blue and normal with golden. **(F)** Differences in the expression of the 24 m^6^A regulators between normal and LUAD tissues. The asterisks show the statistical *p*-value (^**^ *p* < 0.01; ^***^ *p* < 0.001).

Twenty-four m^6^A RNA methylation regulators were selected for LUAD according to previous studies, including 10 writers, 12 readers, and two erasers (**Table S1**). We summarized the dynamic reversible epigenetic modification behavior of these m^6^A regulators and their biological functions in RNA, including mRNA export, mRNA translation, mRNA decay, and mRNA degradation/stability (**Figure 1A**). Somatic mutations in m^6^A regulators were found in 151 (26.63%) of 567 samples. *ZC3H13* had the highest mutation frequency, followed by *KIAA1429* and *IGF2BP1*, while no *CBLL1* mutations were found in the samples (**Figure 1B**). In addition, *YTHDC1* was significantly positively correlated with *ZC3H13*, *YTHDC2*, *FMR1*, and *HNRNPA2B1* (**Supplementary Figure S1B**). There was widespread CNV in the 24 regulatory factors; *METTL16*, *RBM15B*, *METTL14*, *ELAVL1*, and *RBM15* had copy number losses, while *YTHDF1*, *KIAA1429*, *FMR1*, *IGF2BP2*, and *METTL3* showed numerous copy number gains (gene amplification) (**Figure 1C**). The locations of the CNVs in the 24 m^6^A regulators were labeled on the chromosomes (**Figure 1D**). Tumor samples were distinguished from normal samples by three-dimensional PCA (3D-PCA) of the 24 m^6^A regulators. The results showed that the two groups were completely separated from each other (**Figure 1E**). We then compared mRNA expression in normal and tumor samples to explore whether the expression levels of the m^6^A regulators were affected by the above gene variation. Seventeen of the 24 m^6^A regulators showed significant overexpression or downregulation in LUAD samples. *YTHDF1*, *KIAA1429*, *HNRNPC*, and *METTL3* were most significantly upregulated in tumor samples, and *METTL16* and *METTL14* were markedly downregulated (**Figure 1F**). These results showed that the CNV was an important factor in controlling the expression of m^6^A regulators. Most m^6^A regulators underwent remarkable expression changes in LUAD, suggesting that the abnormal status of m^6^A regulators is involved in the development of LUAD.

### Identification of m^6^A Modification Patterns in LUAD

**Figure 2:**
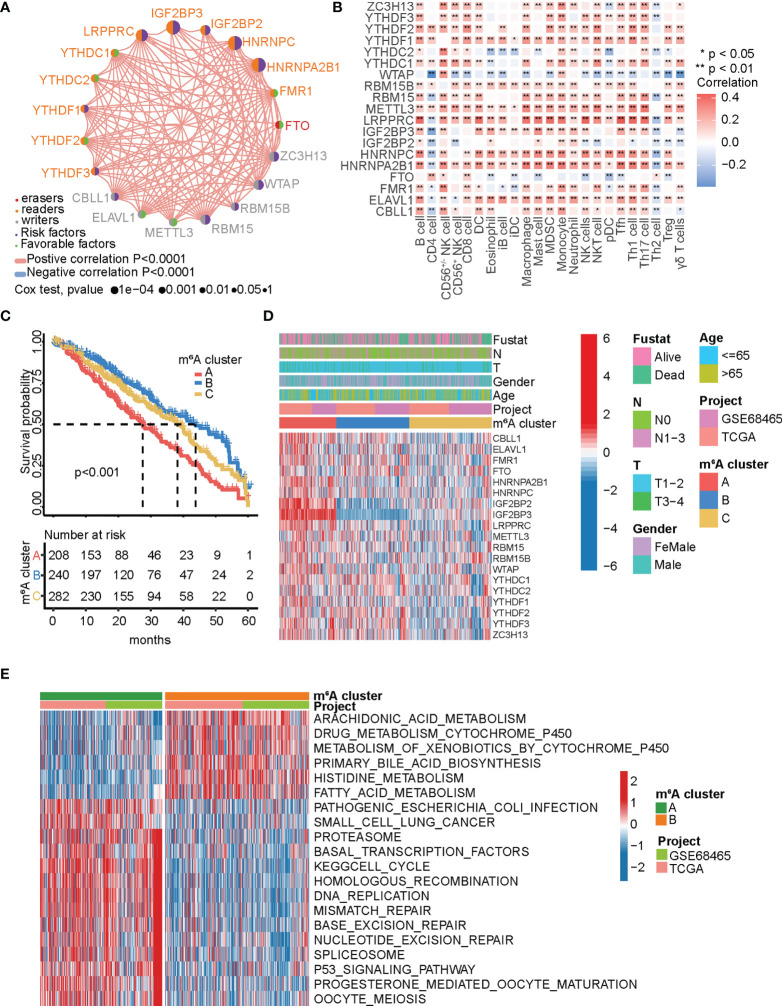
m^6^A methylation patterns and related biological processes. **(A)** The interplay among the m^6^A regulators in LUAD. The m^6^A regulators in three RNA modification types were indicated by the different colors in the circle left. Favorable factors for patients’ survival were indicated by grass green in the circle right and risk factors indicated by blue in the circle right. The circle size indicates the influence of each regulator on prognosis, and the range of values calculated by Log-rank test was represented by the size of each circle. The lines connecting the regulators show their interplay, and the thickness indicates the strength of the association between the regulators. Negative correlation was marked with blue and positive correlation with red. **(B)** Analysis of the relationship between each tumor-infiltrating immune cell type and each m^6^A regulator in LUAD using Spearman’s analysis. Red indicates positive correlation; blue indicates negative correlation. ^*^ *p* < 0.05, ^**^ *p* < 0.01. **(C)** Survival analyses for the three m^6^A modification patterns in from TCGA-LUAD and GSE68645, including 208 cases of m^6^A cluster A, 240 cases of m^6^A cluster B, and 282 cases of m^6^A cluster *C.* Log-rank test, *p* < 0.0001. **(D)** Heatmap showing the correlation between the three m^6^A clusters and the clinicopathological characteristics. Clinicopathological information including age, gender, fustat, and tumor stage, as well as the m^6^A cluster, is shown in annotations above. Red represents high expression, and blue represents low expression. **(E)** Heatmap showing the biological processes in different m^6^A modification patterns obtained by GSVA enrichment analysis. Red shows activated pathways and blue shows inhibited pathways. m^6^A cluster A vs. m^6^A cluster B.

Meta-analysis was performed using TCGA-LUAD and GEO (GSE68465) datasets to further elucidate the modification characteristics of the m^6^A regulators in LUAD. This algorithm finally identified 19 m^6^A regulators through data consolidation. To examine whether the 19 m^6^A regulators could be used as prognostic markers for LUAD, we used univariate Cox regression analysis (**Supplementary Figures S1C** and **S2A–S**). Eight of the 19 genes were significantly correlated with prognosis, including *HNRNPA2B1*, *HNRNPC*, *IGF2BP2*, *IGF2BP3*, *LRPPRC*, *RMB15*, *WTAP*, and *ZC3H13* (HR >1). The prognostic significance of 19 m^6^A regulators in LUAD, the connection to the regulator, and the interactions are shown in the m^6^A regulator network (**Figure 2A**). The results showed a remarkable correlation among writers, readers, and erasers, and this crosstalk was essential for the generation of different m^6^A modification modes. The landscape also suggested that the occurrence and progression of LUAD are related to m^6^A regulators. Effective immune infiltration in tumors is considered a key factor in carcinogenesis and prognosis (50, 51). The correlations between individual regulators and each tumor-infiltrating immune cell type were analyzed using Spearman’s correlation analysis (**Figure 2B**). All m^6^A regulators were significantly correlated with some types of tumor-infiltrating immune cells, suggesting that m^6^A regulators are critical for immune infiltration in tumors. Most m^6^A regulators were significantly positively correlated with immune infiltration, while *WTAP*, an m^6^A methyltransferase, was negatively correlated.

Based on the expression of the 19 m^6^A regulators, the R package “Consensus Cluster Plus” classified patients into various m^6^A modification patterns. Consequently, the unsupervised consensus clustering algorithm revealed that patients were well defined when *k* = 3 (**Supplementary Figure S3A**). Thus, three distinct patient clusters were identified based on m^6^A modification patterns, including 253 cases of cluster A, 324 cases of cluster B, and 359 cases of cluster C. The Kaplan-Meier analysis of the three clusters revealed that cluster A had the worst prognosis, while cluster B had a significant survival advantage (**Figure 2C**). Next, the expression levels of the m^6^A regulators in the three clusters were analyzed; the expression profiles of most m^6^A regulators significantly differed among the three clusters. Cluster A was upregulated compared with the other two clusters, including *CBLL1*, *ELAVL1*, *FMR1*, *HNRNPA2B1*, *HNRNPC*, *IGF2BP2*, *IGF2BP3*, *LRPPRC*, *METTL3*, *RBM15*, *RBM15B*, *WTAP*, *YTHDF1*, and *ZC3H13* (**Supplementary Figure S3B**). The expression levels of *IGF2BP2* and *IGF2BP3* were significantly downregulated in cluster B but upregulated in cluster A (**Figure 2D**). These results indicate that *IGF2BP2* and *IGF2BP3* are the dominant risk factors for malignant progression. Based on the KEGG gene set, GSVA enrichment analysis was used to explore the biological processes of the two m^6^A modification patterns (**Figure 2E**; **Supplementary Figures S3C, D** **)**. We found that cluster A was clearly enriched in the process of DNA repair, such as nucleotide excision repair, mismatch repair, DNA replication, and base excision repair; cluster B was prominently enriched in metabolism-associated pathways, such as fatty acid metabolism, amino acid metabolism, arachidonic metabolism, drug metabolism cytochrome P450, and primary bile acid biosynthesis. Cluster C was enriched in pathways associated with immune activity, including intestinal immune network production, antigen processing and presentation, and cytokine receptor interaction.

### Immune Landscapes With Distinct m^6^A Modification Patterns

**Figure 3:**
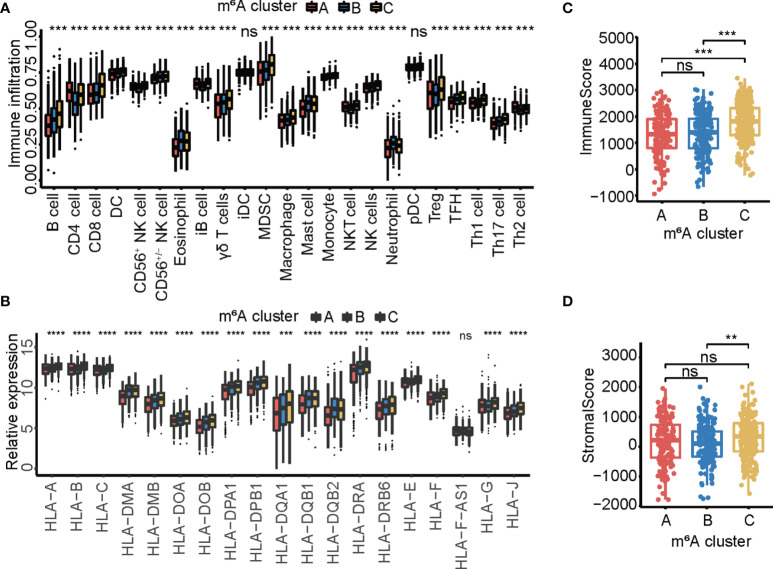
The immune landscape in three m^6^A modification patterns. **(A)** The relative abundance of 23 tumor-infiltrating immune cell types in three m^6^A modification patterns. The upper and lower ends of the boxes represented interquartile range of values. The lines in the boxes represented median value, and black dots showed outliers. The asterisks represented the statistical *p*-value (^**^ *p* < 0.01; ^***^ *p* < 0.001). **(B)** The expression of MHC molecules in three m^6^A modification patterns. The upper and lower ends of the boxes represented interquartile range of values. The lines in the boxes represented median value, and black dots showed outliers. The asterisks represented the statistical *p*-value (ns *p* > 0.05; ^**^ *p* < 0.01; ^***^ *p* < 0.001). **(C)** Box plot showing the immune scores of the three m^6^A clusters. **(D)** Box plot showing the stromal score of the three m^6^A clusters.

Subsequently, the correlation between 23 tumor-infiltrating immune cell types and three m^6^A cluster subsets was examined by ssGSEA analysis. The results showed that cluster C was associated with more adaptive and innate immune cell infiltration, including CD8 cells, NK cells, Tregs, MDSCs, macrophages, and B cells (**Figure 3A**). Consistently, cluster C exhibited a comprehensively elevated expression of MHC molecules (**Figure 3B**). Immune cell infiltration in the three m^6^A cluster subsets was assessed using the ESTIMATE algorithm. The results showed that cluster C exhibited higher immune and stromal scores, indicating that cluster C had significantly increased immune cell infiltration (**Figures 3C, D** **)**, which was mainly due to increased immune infiltration and high MHC expression.

### m^6^A Phenotype-Related DEGs in LUAD

**Figure 4:**
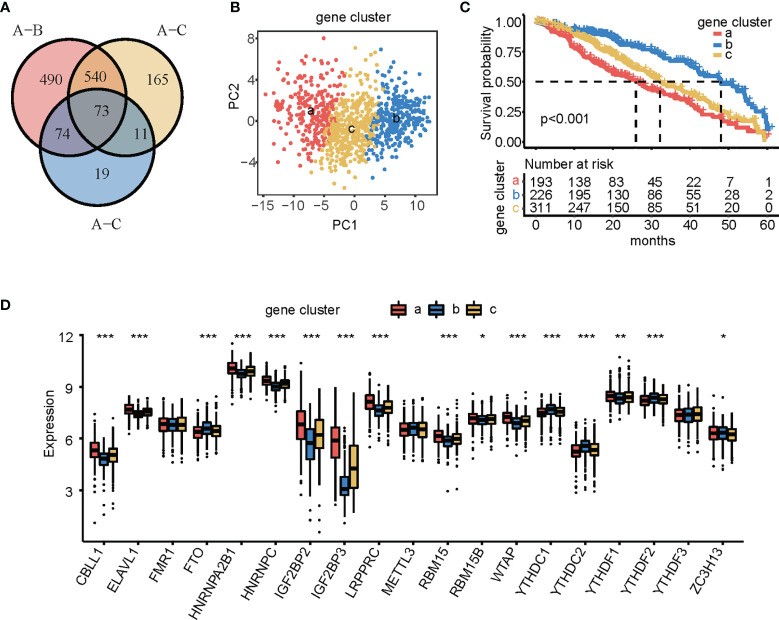
Identification of m^6^A phenotype-related differentially expressed genes (DEGs) and construction of gene clusters. **(A)** Venn diagram showing 73 m^6^A phenotype-related DEGs between three m^6^A clusters. **(B)** Principal component analysis of three gene cluster patterns. **(C)** Kaplan-Meier curves showing the overall survival of the patients in the three gene clusters, including 193 cases of gene cluster a, 226 cases of gene cluster b, and 311 cases of gene cluster c. Log-rank test, *p* < 0.001. **(D)** Expression of the 24 m^6^A regulators in the three gene clusters. The upper and lower ends of the boxes represented interquartile range of values. The lines in the boxes represented median value, and black dots showed outliers. The asterisks represented the statistical *p*-value (^*^ *p* < 0.05; ^**^ *p* < 0.01; ^***^ *p* < 0.001).

To further elucidate the underlying biological processes and functional annotation of a single m^6^A modification pattern, seventy-three m^6^A phenotype-related DEGs were identified using the R package “Limma” and represented on a Venn diagram (**Figure 4A**). GO enrichment analysis for DEGs was performed using the R package “Cluster Profiler.” Surprisingly, these genes were remarkably related to nuclear division and organelle fission (**Supplementary Figure S3E**). To further validate this regulatory mechanism, we obtained 68 prognosis-related DEGs using univariate Cox regression analysis. Unsupervised cluster analysis was performed on the 68 prognosis-related DEGs, which were divided into three subgroups: gene clusters a, b, and c. We performed PCA on the expression of the 68 DEGs, showing that the three groups were completely separated from each other (**Figure 4B**). The results showed 226, 193, and 311 patients in gene clusters a, b, and c, respectively. Patients in gene cluster a were associated with a worse prognosis, patients in gene cluster b had a better prognosis, and patients in gene cluster c had a moderate prognosis (**Figure 4C**). m^6^A regulator expression was significantly different between the three gene clusters, which was the same as that of the m^6^A modification patterns (**Figure 4D**).

### Construction of an m^6^A Score Model

**Figure 5:**
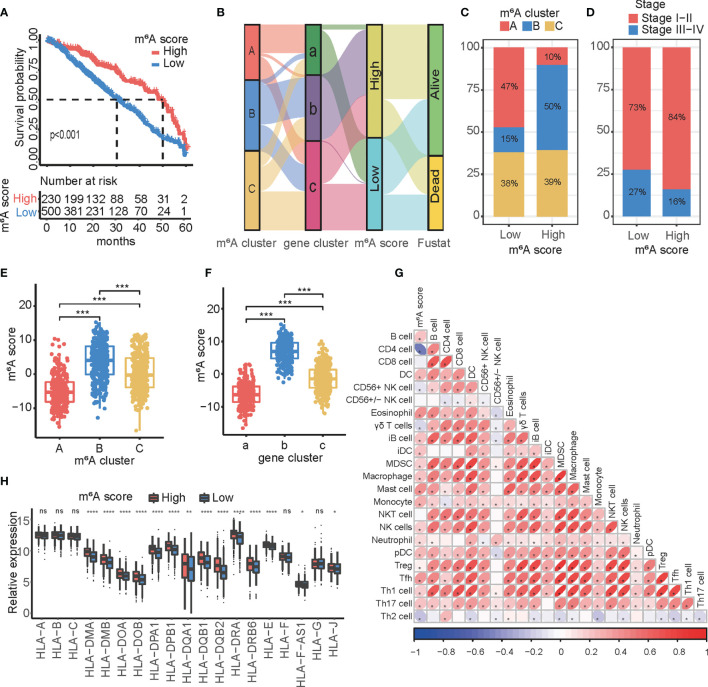
Construction and characteristics of the m^6^A score. **(A)** Kaplan-Meier curves showing the differences in survival of the high (*n* = 230) and low (*n* = 500) m^6^A score groups in LUAD. Log-rank test, *p* < 0.001. **(B)** Changes in m^6^A clusters among groups with different gene clusters, m^6^A score, and fustat (alive, dead) are shown through an alluvial diagram. **(C)** The proportion of the three m^6^A modification patterns in high and low m^6^A score groups. **(D)** The proportion of patients with different stages in high and low m^6^A score groups. **(E)** Differences in the m^6^A score between the three m^6^A clusters. The asterisks represented the statistical *p*-value (^***^ *p* < 0.001). **(F)** Differences in m^6^A score between the three gene clusters. The asterisks represented the statistical *p*-value (^***^ *p* < 0.001). **(G)** Correlations between the m^6^A score and tumor-infiltrating immune cells using Spearman’s analysis. The positive and negative correlations are marked with red and blue, respectively. **(H)** Differences in the expression of MHC molecules between the high and low m^6^A score groups. The upper and lower ends of the boxes represented interquartile range of values. The lines in the boxes represented median value, and black dots showed outliers. The asterisks represented the statistical *p* value (ns p > 0.05; ^*^ *p* < 0.05; ^**^ *p* < 0.01; ^****^ *p* < 0.0001).

To predict the immune status and prognosis in a single patient, we sought to develop an m^6^A score based on the 68 DEGs identified. Therefore, patients were divided into high- or low-m^6^A score groups based on the cutoff value. The high m^6^A score group had a better clinical survival profile (**Figure 5A**). In addition, we externally verified m^6^A score model to predict the prognosis of patients with lung adenocarcinoma from GSE11969, GSE13213, GSE37745, and GSE50081 datasets (**Supplementary Figures S4A–D**). An alluvial diagram was used to illustrate the workflow of the m^6^A score construction and to visualize the attribute changes in individual patients (**Figure 5B**). The results indicated that gene cluster b was associated with a high m^6^A score, whereas gene cluster c was associated with a lower m^6^A score. Notably, most patients who were still alive were included in the high m^6^A score group. Most patients with m^6^A cluster A were defined as low m^6^A, while patients with m^6^A cluster B had a high m^6^A score. The m^6^A cluster C was similarly distributed (**Figure 5C**). The group with a statistically low m^6^A score had more advanced patients (**Figure 5D**). We also examined the relationship between the above subtypes and the m^6^A score. Kruskal−Wallis analysis revealed that m^6^A cluster B and gene cluster b exhibited the highest m^6^A score, while m^6^A cluster A and gene cluster a showed the lowest score (**Figures 5E, F** **)**. Multimodel crossvalidation suggested that the m^6^A score could serve as a prediction model for LUAD. Based on Spearman’s analysis, a heat map demonstrated a positive correlation between the m^6^A score and tumor-infiltrating immune cells (**Figure 5G**). Furthermore, the high m^6^A score group also exhibited a comprehensively elevated expression of MHC molecules (**Figure 5H**). These results indicated that the m^6^A score may be used as a model to predict the immune status in LUAD.

### Characteristics of Tumor Somatic Mutations in Patients From the High and Low m^6^A Score Groups

**Figure 6:**
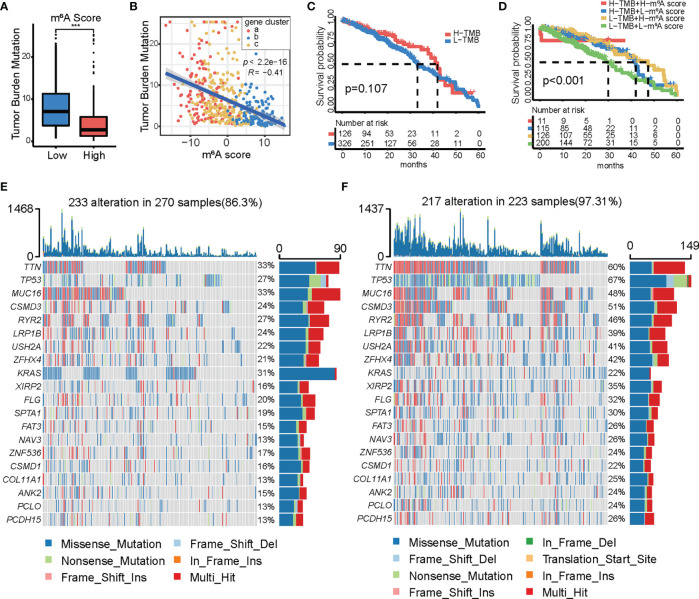
Characteristics of m^6^A modification in tumor mutation burden (TMB). **(A)** Differences in TMB distribution between the high and low m^6^A score groups. **(B)** Correlation between TMB and m^6^A score. **(C)** Kaplan-Meier curves showing the differences in survival between the high (*n* = 126) and low (*n* = 326) TMB groups. Log-rank test, *p* = 0.107. **(D)** Survival analyses for subgroup patients stratified by both m^6^A score and TMB using Kaplan-Meier curves. H, high; L, low. TMB, tumor mutation burden. Log-rank test, *p* < 0.001. **(E, F)** Waterfall plot of tumor somatic mutations in patients with high **(E)** and low **(F)** m^6^A score. Each column represents each individual patient. The upper bar plot represents TMB. The number on the right represents the mutation frequency in each regulator. The bar graph on the right shows the proportion of each variant type. The stacked bar chart below shows the conversion of each sample.

Several studies have confirmed correlations between tumor somatic mutations, genomic alterations, and immunotherapeutic effects. Therefore, we evaluated the distribution of the tumor mutation burden (TBM) in different m^6^A score groups. A box and scatter diagram showed that the high m^6^A score group had a lower TMB and that the m^6^A score was negatively correlated with TMB (**Figures 6A, B** **)**. K-M survival analysis showed that TMB alone was not sufficient to accurately predict the prognosis of LUAD (**Figure 6C**). To further understand the relationship between TMB, m^6^A score, and survival outcomes, a K-M survival analysis based on the combination of m^6^A score and TMB was performed. The results revealed that the low TMB and low m^6^A score subgroups were associated with poor prognosis. The combination of the m^6^A score and TMB could accurately predict the quality of life of patients with LUAD (**Figure 6D**). These data emphasize the impact of the m^6^A score and TMB on cancer development. A list of the somatic mutations in the high- and low-m^6^A score groups is shown (**Figures 6E, F** **)**. The low m^6^A score group presented more extensive TMB than the high m^6^A score group, except for KRAS (22% vs. 31%).

### Strong Association of the m^6^A Score With Clinicopathological Characteristics in LUAD

**Figure 7:**
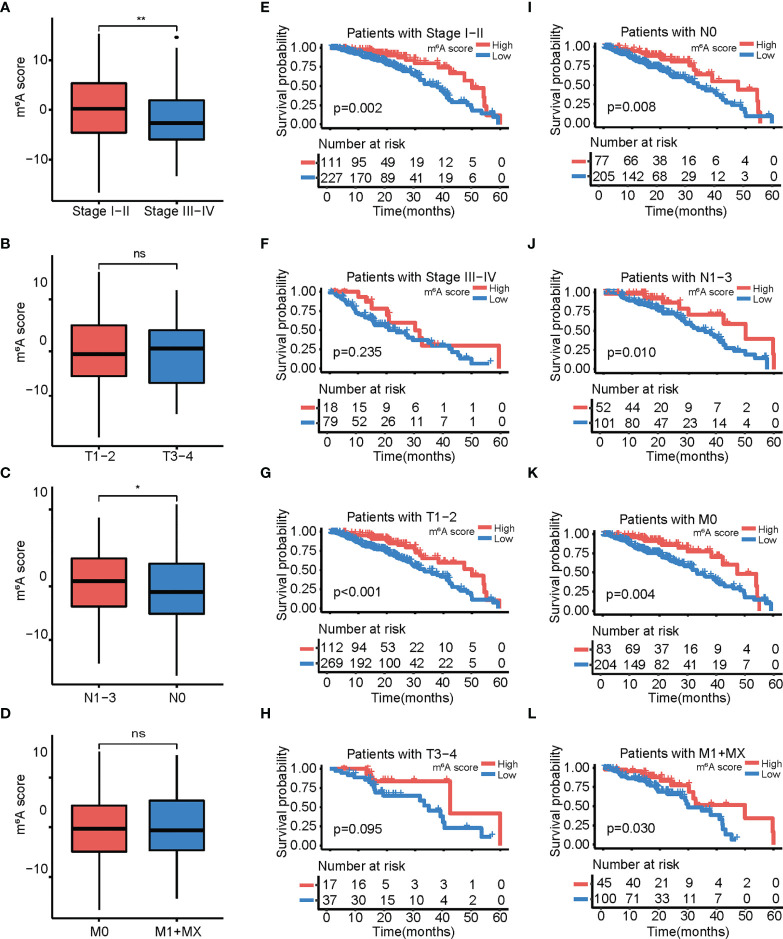
Relationship between the m^6^A score and different clinical characteristics. **(A−D)** Box plot showing differences in m^6^A score among patients with different clinical characteristics. **(A)** Stages I–II vs. stages III–VI; **(B)** T 1–2 vs. T 3–4; **(C)** N 1–3 vs. N 0; **(D)** M 0 vs. M 1+X. **(E−I)** Kaplan-Meier curves showing the differences in survival depending on the m^6^A score and different clinical characteristics. **(E)** Stages I–II. **(F)** Stages III–VI; **(G)** T 1–2; **(F)** T 3–4; **(I)** N 0; **(J)** N 1–3; **(K)** M 0; **(L)** M 1+X. The asterisks represented the statistical p-value (ns *p* > 0.05; *p <0.05; **p < 0.01).

We examined the relationship between the m^6^A score and clinicopathological characteristics, and observed that the m^6^A score significantly differed between patients by stage, node (N), but not tumor (T) and metastasis (M) (**Figures 7A–D**). In this study, the prognostic value of the m^6^A score was determined for different clinicopathological characteristics, and it was found that patients with high m^6^A score had significantly longer overall survival than patients with low m^6^A score in stages I/II, T1/2, N0, and M0 (**Figures 7E–L**). Therefore, we considered the m^6^A score to be a possible prognostic factor for LUAD.

### The Role of the m^6^A Score in Predicting Benefits From Radiotherapy and Immunotherapy

**Figure 8:**
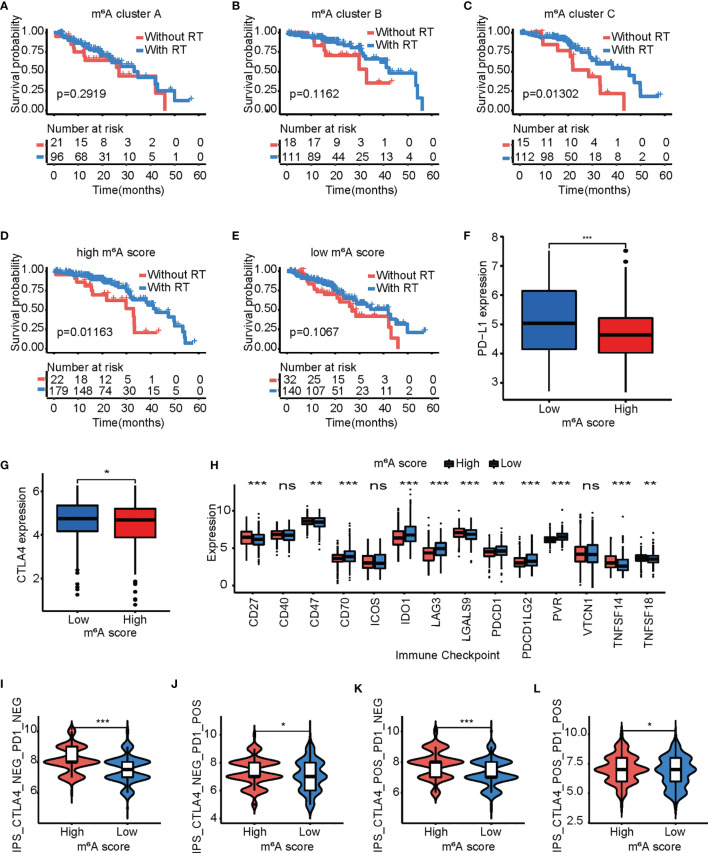
The m^6^A score model predicts the benefits of radiotherapy and immunotherapy. **(A–C)** Kaplan-Meier curves of overall survival for patients in m^6^A cluster A **(A)**, m^6^A cluster B **(B)**, and m^6^A cluster C **(C)** based on acceptance/rejection of radiotherapy. Log-rank test. **(D, E)** Kaplan-Meier curves of overall survival for patients with a high **(D)**/low **(E)** m^6^A score based on acceptance/rejection of radiotherapy. Log-rank test. **(F, G)** Comparison of the PD-1 **(F)**/CTLA4 **(G)** expression levels between the high and low m^6^A score groups. Log-rank test. (ns *p* > 0.05;^*^ *p* < 0.05; ^**^ *p* < 0.01; ^***^ *p* < 0.001). **(H)** Differences in the expression of other immune checkpoints between the high and low m^6^A score groups. Log-rank test. (^*^ *p* < 0.05; ^**^ *p* < 0.01; ^***^ *p* < 0.001). **(I–L)** Box plot representing the relative distribution of the immunophenoscore between the low and high m^6^A score groups in LUAD patients based on TCIA database, **(I)** CTLA4^−^ PD1^−^; **(J)** CTLA4^−^ PD1^+^; **(K)** CTLA4^+^ PD1^−^; and **(L)** CTLA4^+^ PD1^+^. The asterisks represented the statistical p-value (*p < 0.05; ***p < 0.001).

The prognostic value of the m^6^A score was investigated for patients with LUAD who accepted radiotherapy. Surprisingly, only in the m^6^A cluster C group did patients with radiotherapy had a better quality of life than those without radiotherapy (**Figures 8A–C**). Interestingly, patients with radiotherapy had a better quality of life than those without radiotherapy in the high m^6^A score group. The survival advantage from radiotherapy in the low m^6^A score group was virtually zero (**Figures 8D, E**).

Furthermore, the expression levels of PD-1 and CTLA4 were examined, and a remarkable elevation was observed in the low m^6^A score group (**Figures 8F, G** **)**. The levels of other immune checkpoints were then compared in the high and low m^6^A score groups. The high m^6^A score group exhibited higher expression of *CD27*, *CD28*, *LGALS9*, *TNFSF14*, and *TNFSF18*, while the low m^6^A score group had higher expression of *CD70*, *IDO1*, *LAG3*, *PDCD1LG2*, and *PVR* (**Figure 8H**). In addition to the well-known TMB and checkpoint, IPS is one of the newly discovered predictive factors that is widely used and is strongly suggested to assess patients’ reaction to immunotherapy (52, 53). Compared with patients with low m^6^A score, patients with high m^6^A score exhibited significant clinical benefits from anti-PD-1/CTLA4 immunotherapy (**Figures 8I–L**). These findings confirmed that the levels of tumor m^6^A modification modes play a significant role in the regulation of the expression of immune molecules.

## Discussion

m^6^A methylation, the most common form of mRNA modification, plays an indispensable role in posttranscriptional regulation. In recent years, increasing studies have demonstrated the importance of m^6^A modification in congenital immunity and inflammation, and its antitumor effects through coaction with unequal m^6^A regulators (54–56). Many studies have focused on single m^6^A regulators or tumor-infiltrating immune cell types; however, the association between overall tumor microenvironment characteristics and integrated m^6^A regulators remains poorly understood. Therefore, the distinction of inverse m^6^A modification modes in tumors will contribute to understanding the relationship between m^6^A regulators and the antitumor immune response. Here, we constructed a prognostic model for effective therapeutic strategies.

Based on the presentation level of 21 m^6^A regulators, this study revealed three m^6^A modification modes with different characteristics. The m^6^A cluster A was characterized by a poor prognosis and enrichment in the process of DNA repair. The m^6^A cluster B was characterized by a favorable prognosis and enrichment in metabolism-associated pathways. The m^6^A cluster C was characterized by activation of the immune system and a higher stromal cell score. Previous studies have reported that the immune microenvironment plays a key role in tumor evolution and immunotherapy (57). The characteristics of tumor immune infiltration, including the activity of CD4 and CD8 T cells, macrophages, and natural killer cells, are associated with immunotherapeutic efficacy (58–60). Here, we verified that the m^6^A cluster C was significantly associated with elevated immune cell permeation and high stromal cell infiltration. Previous studies demonstrated that tumors with immune-excluded phenotype showed the presence of abundant immune cells, whereas these immune cells do not penetrate the parenchyma but instead are retained in the stroma that surrounds nests of tumor cells (61). Moreover, stromal cells affect the killing effect of IL-12 delivery, empowering CAR-T immune infiltrating cells (62). Thus, it was not surprising to find that m^6^A cluster C aroused congenital immunity but a poor prognosis.

DEGs that discerned disparate m^6^A modification patterns were deemed to be m^6^A phenotype-related gene signatures. Parallel to the m^6^A clustering construction, three gene cluster types were constructed according to the m^6^A-related DEGs, which were significantly related to distinct clinical outcomes and landscapes of immune infiltration. These findings suggest that m^6^A modification is involved in tumorigenesis, tumor development, and immune cell infiltration. To qualify the m^6^A modification patterns of individual samples, a quantitative model named “the m^6^A score” was constructed. As a result, m^6^A cluster B and gene cluster b exhibited higher m^6^A score, while m^6^A cluster A and gene cluster a exhibited a lower m^6^A score. We found that the m^6^A score was positively associated with immune cell infiltration. Surprisingly, the high m^6^A score group also exhibited elevated expression of MHC molecules and lower expression of PD-1 and CTLA4.

Our analysis also demonstrated an obvious subtractive association between the m^6^A score and TMB. Unlike the results of previous studies, there was no disparity in survival between the high and low TMB groups in LUAD, while the incorporation of the m^6^A score and TMB level could refine the clinical outcomes of patients with LUAD. We also confirmed that the m^6^A score could be used to evaluate the clinicopathological characteristics of patients involving the clinical stage. Exhaustive associations between the m^6^A score and clinicopathological characteristics could be discovered in this study. Similarly, the m^6^A score could play a role as a stand-alone prognostic biomarker for prognosis. A high m^6^A score and the m^6^A cluster C subgroup were beneficial to radiotherapy and anti-PD-1/CTLA4 immunotherapy, which was due to increased immune cell infiltration and immunocompetence. The m^6^A score model could predict the power of adjuvant radiotherapy and the clinical effect of the patient type on the response to anti-PD-1/CTLA4 immunotherapy. These findings provide new insights into the relationship between tumor-infiltrating immune cells and cancer immunotherapy, and increase our capacity to select clinical immunotherapy strategies.

We compiled a list of 24 identified m^6^A regulators; however, newly recognized regulators must be integrated into the model to achieve the highest precision with the m^6^A modification patterns. Furthermore, the m^6^A modification patterns and m^6^A score were distinguished by employing retrospective data collection; therefore, a future cohort of patients with LUAD accepting immunotherapy is required to confirm our findings. In addition, since immunotherapy showed strong clinical advantages in a fraction of patients with high m^6^A score, more clinical cases and tumor types should be introduced into the predicted models to increase precision.

## Conclusion

In conclusion, we systematically analyzed m^6^A modification patterns among 936 LUAD samples considering 24 m^6^A regulators, and comprehensively evaluated their prognostic value and correlation with tumor-infiltration immune cell characteristics. The comprehensive analysis of individual tumor m^6^A modification patterns will greatly enhance our understanding of the tumor microenvironment and the characterization of immune cell infiltration. This study provides a basis for improving current immune therapies and promoting the clinical success of immunotherapy. However, due to the small number of samples and the clinical heterogeneity of the study cohort, large-scale cohort studies and prospective studies are necessary to verify the predictive value of the m^6^A score in the clinical treatment and prognosis of LUAD.

## Data Availability Statement

The datasets presented in this study can be found in online repositories. The names of the repository/repositories and accession number(s) can be found in the article/**Supplementary Material**.

## Ethics Statement

The studies involving human participants were reviewed and approved by the Medical Ethics Committee of Guangzhou Medical University Tumor Hospital. The patients/participants provided their written informed consent to participate in this study. The animal study was reviewed and approved by the Medical Ethics Committee of Guangzhou Medical University Tumor Hospital. Written informed consent was obtained from the individual(s) for the publication of any potentially identifiable images or data included in this article.

## Author Contributions

MZ and YC have contributed equally to this work. MZ, YL, XZ, and QY designed the experiments. MZ, YL, and QY wrote the paper. MZ, YC, QM, JZ, TZ, YX, ZW, and DS analyzed the data. All authors contributed to the article and approved the submitted version.

## Funding

This study was supported by the National Natural Science Foundation of China (grant numbers 81870409, to QY) and Guangzhou Key Medical Discipline Construction Project (to QY).

## Conflict of Interest

The authors declare that the research was conducted in the absence of any commercial or financial relationships that could be construed as a potential conflict of interest.

## Publisher’s Note

All claims expressed in this article are solely those of the authors and do not necessarily represent those of their affiliated organizations, or those of the publisher, the editors and the reviewers. Any product that may be evaluated in this article, or claim that may be made by its manufacturer, is not guaranteed or endorsed by the publisher.

## Supplementary Material

Supplementary Figure S1 The correlation and prognostic analysis of 19 m^6^A regulators. **(A)** Overview of this analysis. **(B)** Correlation analyses for 19 m^6^A regulators. Red indicates positive correlation; blue indicates negative correlation. (*P < 0.05; **P < 0.01; ***P < 0.001). **(C)** Prognostic analyses for 19 m^6^A regulators using univariate Cox regression analysis. Hazard ratio >1 indicated risk factors for survival and represent by red. hazard ratio <1 indicated risk factors for survival and represented by blue.

Supplementary Figure S2 Prognostic analysis of 19 m^6^A regulators. **(A–S)**. Kaplan-Meier survival curves are used to analyze the survival difference between high and low gene expression groups of 19 m^6^A regulators. High gene expression groups indicated by red. Low gene expression groups indicated by blue.

Supplementary Figure S3 Consensus clustering of 24 m^6^A regulators in lung adenocarcinoma. **(A)** Consensus clustering matrix for k = 3. **(B)** Expression of the 24 m^6^A regulators in the three m^6^A clusters. The upper and lower ends of the boxes represented interquartile range of values. The lines in the boxes represented median value, and black dots showed outliers. The asterisks represented the statistical p value (*P< 0.05; **P< 0.01; ***P< 0.001). **(C, D**) Heatmap showing the biological processes in different m^6^A modification patterns obtained by GSVA enrichment analysis. Red shows activated pathways and blue shows inhibited pathways. B m^6^A cluster A vs m^6^A cluster C; C m^6^A cluster B vs m^6^A cluster C. **(E)** GO enrichment analysis of the 73 m^6^A phenotype-related DEGs.

Supplementary Figure S4 External validation of the m^6^A score model in lung adenocarcinoma patients from GSE11969, GSE13213, GSE37745, and GSE50081 datasets. **(A)** Kaplan-Meier curves showing the differences in survival of the high and low m^6^A score groups in GSE11969 (P = 0.002, Log-rank test). **(B)** Kaplan-Meier curves showing the differences in survival of the high and low m^6^A score groups in GSE13213 (P = 0.002, Log-rank test). **(C)** Kaplan-Meier curves showing the differences in survival of the high and low m^6^A score groups in GSE37745 (P = 0.032, Log-rank test). **(D)** Kaplan-Meier curves showing the differences in survival of the high and low m^6^A score groups in GSE50081 (P < 0.001, Log-rank test).

## Glossary

m^6^A: N6-methyladenosine
LUAD: lung adenocarcinoma
*METTL3*: methyltransferase-like 3
*METTL14*: methyltransferase-like 14
*METTL16*: methyltransferase-like16
*ZC3H13*: zinc finger CCCH domain-containing protein 13
*ELAVL1*: ELAV-like RNA binding protein 1
*CBLL1*: Cbl proto-oncogene-like 1
*RBM15*: RNA-binding motif protein 15
*RBM15B*: RNA-binding motif protein 15B
*WTAP*: WT1-associated protein
*KIAA1429*: VIR-like m^6^A methyltransferase associated
*FTO*: fat mass and obesity-associated protein
*ALKBH5*: AlkB homolog H5
*YTHDF1*: YTH domain family 1
*YTHDF2*: YTH domain family 2
*YTHDF3*: YTH domain family 3
*YTHDC1*: YTH domain containing 1
*YTHDC2*: YTH domain containing 2
*IGF2BP1*: IGF2 mRNA-binding proteins 1
*IGF2BP2*: IGF2 mRNA-binding proteins 2
*IGF2BP3*: IGF2 mRNA-binding proteins 3
*FMR1*: Fragile X mental retardation 1
*LRPPRC*: leucine-rich pentatricopeptide repeat containing
*HNRNPC*: heterogeneous nuclear ribonucleoprotein C
*HNRNPA2B1*: heterogeneous nuclear ribonucleoprotein A2/B1
NMF: nonnegative matrix factorization
ssGESA: single sample GSEA
PCA: principal component analysis
TME: tumor microenvironment
TMB: tumor mutation burden
ICIs: immune checkpoint inhibitors
TCGA: The Cancer Genome Atlas
GEO: Gene Expression Omnibus
GSVA: gene set variation analysis
DEGs: differentially expressed genes
HR: hazard ratios
CNV: copy number variation
3D-PCA: three-dimensional principal component analysis
KEGG: Kyoto Encyclopedia of Genes and Genomes
MHC: major histocompatibility complex
MSI: microsatellite instability
IPS: immunophenoscore
B cell: activated B cell
CD4 cell: activated CD4 T cell
CD8 cell: activated CD8 T cell
DC: activated dendritic cell
CD56+ NK cell: CD56bright natural killer cell
CD56+/− NK cell: CD56dim natural killer cell
eosinophil: eosinophil
γδ T cells: gamma delta T cell
iB cell: immature B cell
iDC: immature dendritic cell
NKT cell: natural killer T cell
NK cells: natural killer cell
pDC: plasmacytoid dendritic cell
Treg: regulatory T cell
Tfh: T follicular helper cell
Th1 cell: T helper 1 cell
Th2 cell: T helper 2 cell
Th17 cell: T helper 17 cell
