# Supplementary figures and images for: Characterization of m6A RNA Methylation Regulators Predicts Survival and Immunotherapy in Lung Adenocarcinoma
Source: Front Immunol. 2021 Dec 17;12:782551. doi: 10.3389/fimmu.2021.782551 (PMC8718692; doi:10.3389/fimmu.2021.782551)

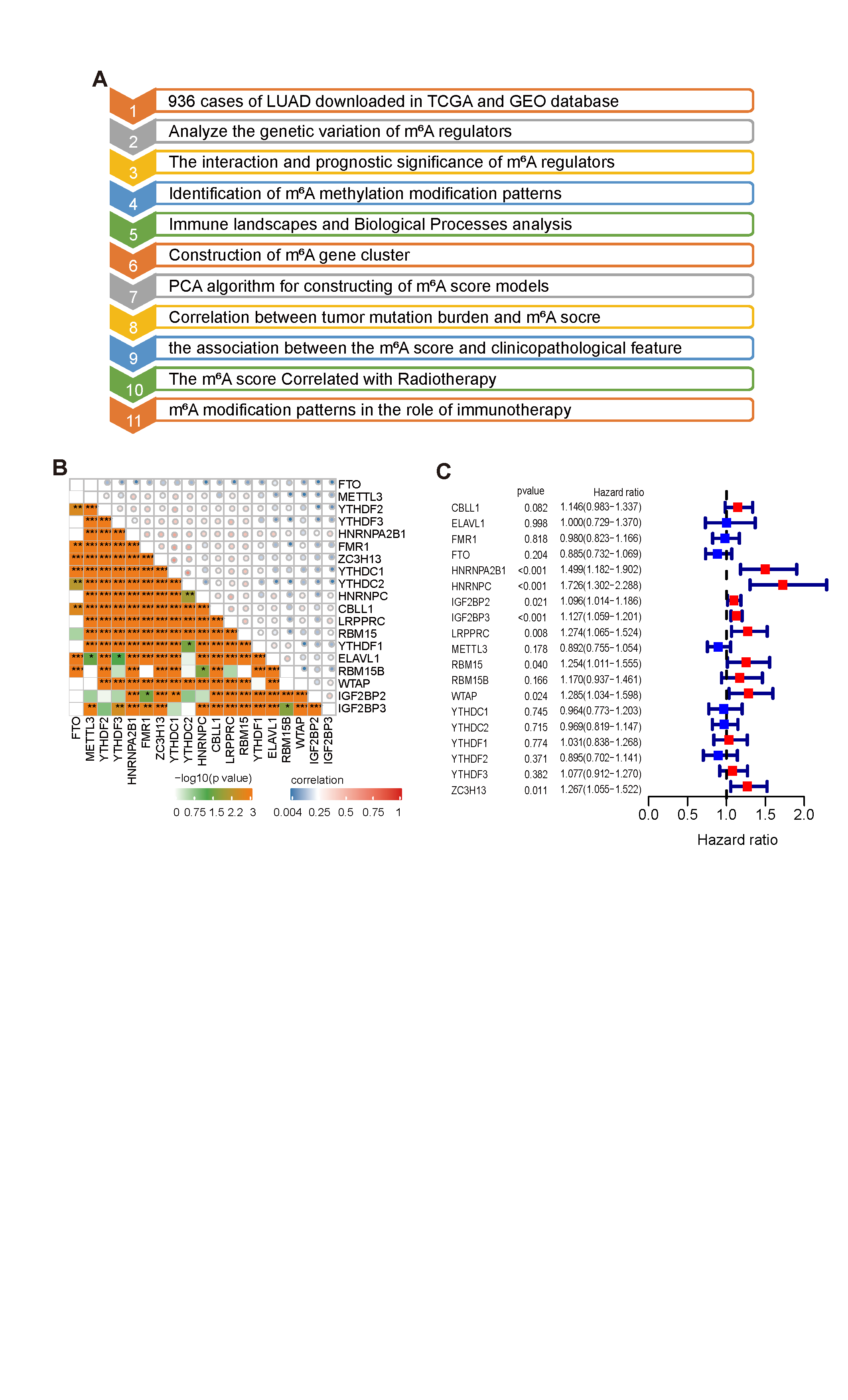

Supplement: Supplementary Figure S1 — The correlation and prognostic analysis of 19 m6A regulators. (A) Overview of this analysis. (B) Correlation analyses for 19 m6A regulators. Red indicates positive correlation; blue indicates negative correlation. (*P < 0.05; **P < 0.01; ***P < 0.001). (C) Prognostic analyses for 19 m6A regulators using univariate Cox regression analysis. Hazard ratio >1 indicated risk factors for survival and represent by red. hazard ratio <1 indicated risk factors for survival and represented by blue. [file Image_1.tiff]

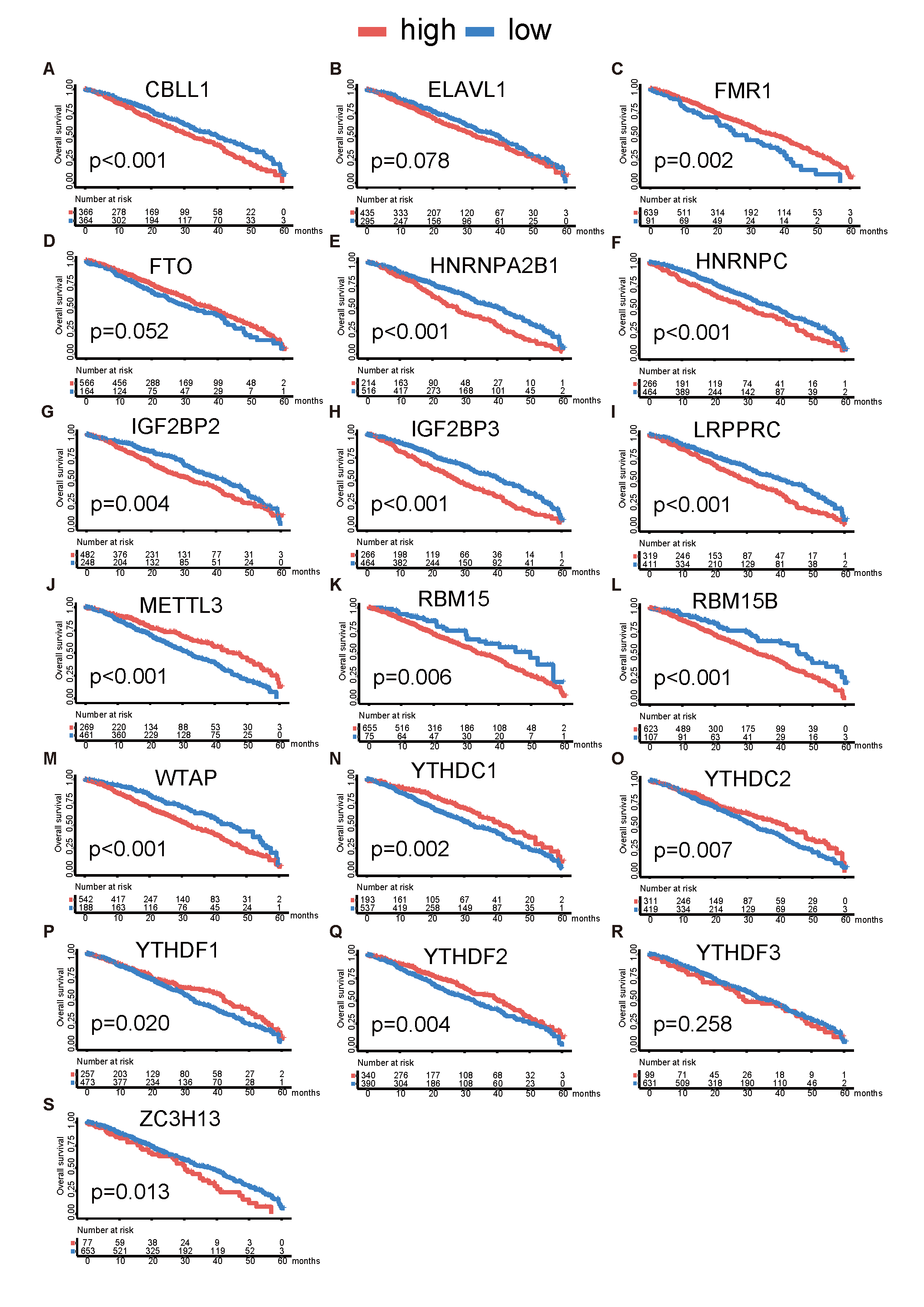

Supplement: Supplementary Figure S2 — Prognostic analysis of 19 m6A regulators. (A–S). Kaplan-Meier survival curves are used to analyze the survival difference between high and low gene expression groups of 19 m6A regulators. High gene expression groups indicated by red. Low gene expression groups indicated by blue. [file Image_2.tif]

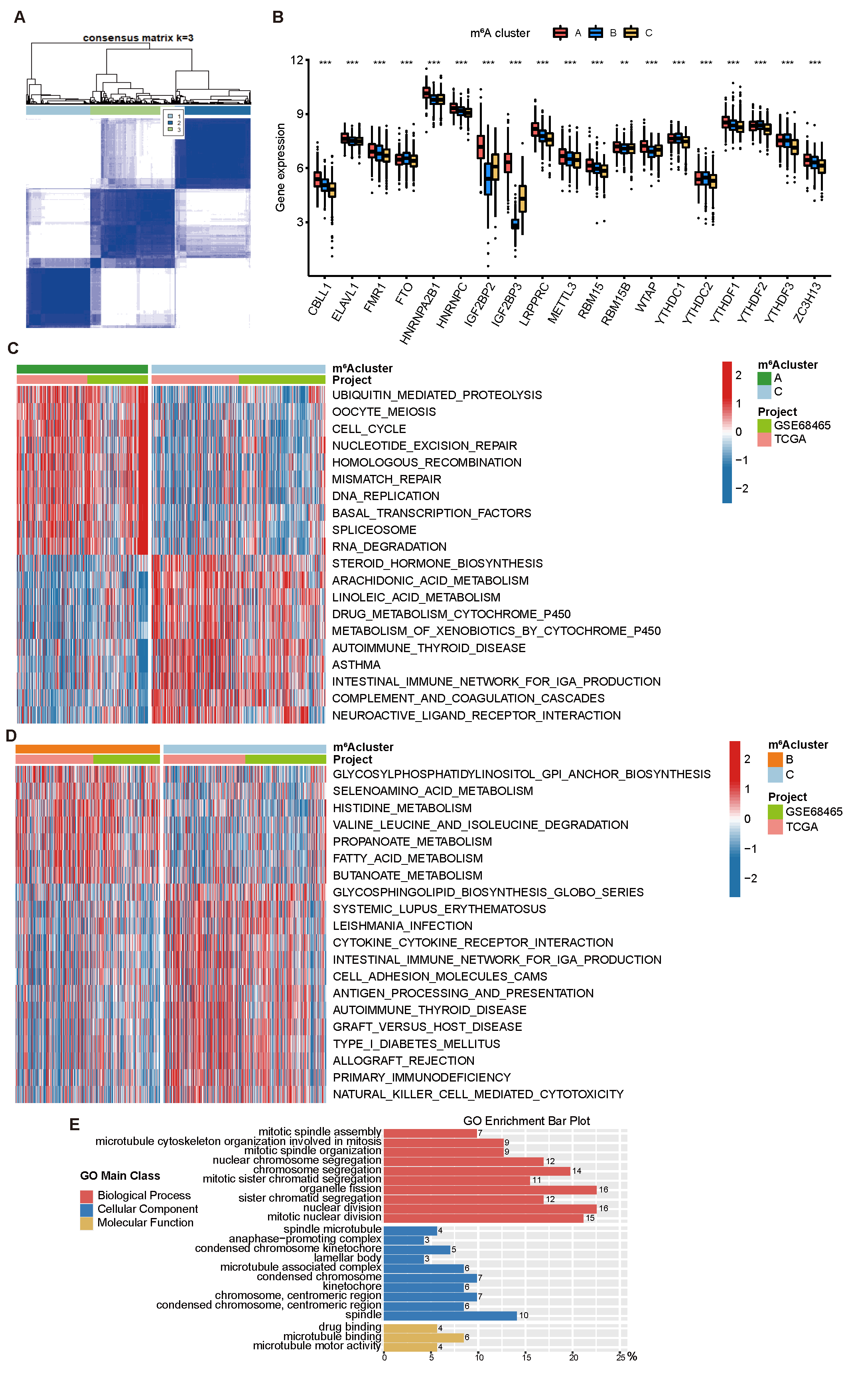

Supplement: Supplementary Figure S3 — Consensus clustering of 24 m6A regulators in lung adenocarcinoma. (A) Consensus clustering matrix for k = 3. (B) Expression of the 24 m6A regulators in the three m6A clusters. The upper and lower ends of the boxes represented interquartile range of values. The lines in the boxes represented median value, and black dots showed outliers. The asterisks represented the statistical p value (*P< 0.05; **P< 0.01; ***P< 0.001). (C, D) Heatmap showing the biological processes in different m6A modification patterns obtained by GSVA enrichment analysis. Red shows activated pathways and blue shows inhibited pathways. B m6A cluster A vs m6A cluster C; C m6A cluster B vs m6A cluster C. (E) GO enrichment analysis of the 73 m6A phenotype-related DEGs. [file Image_3.tif]

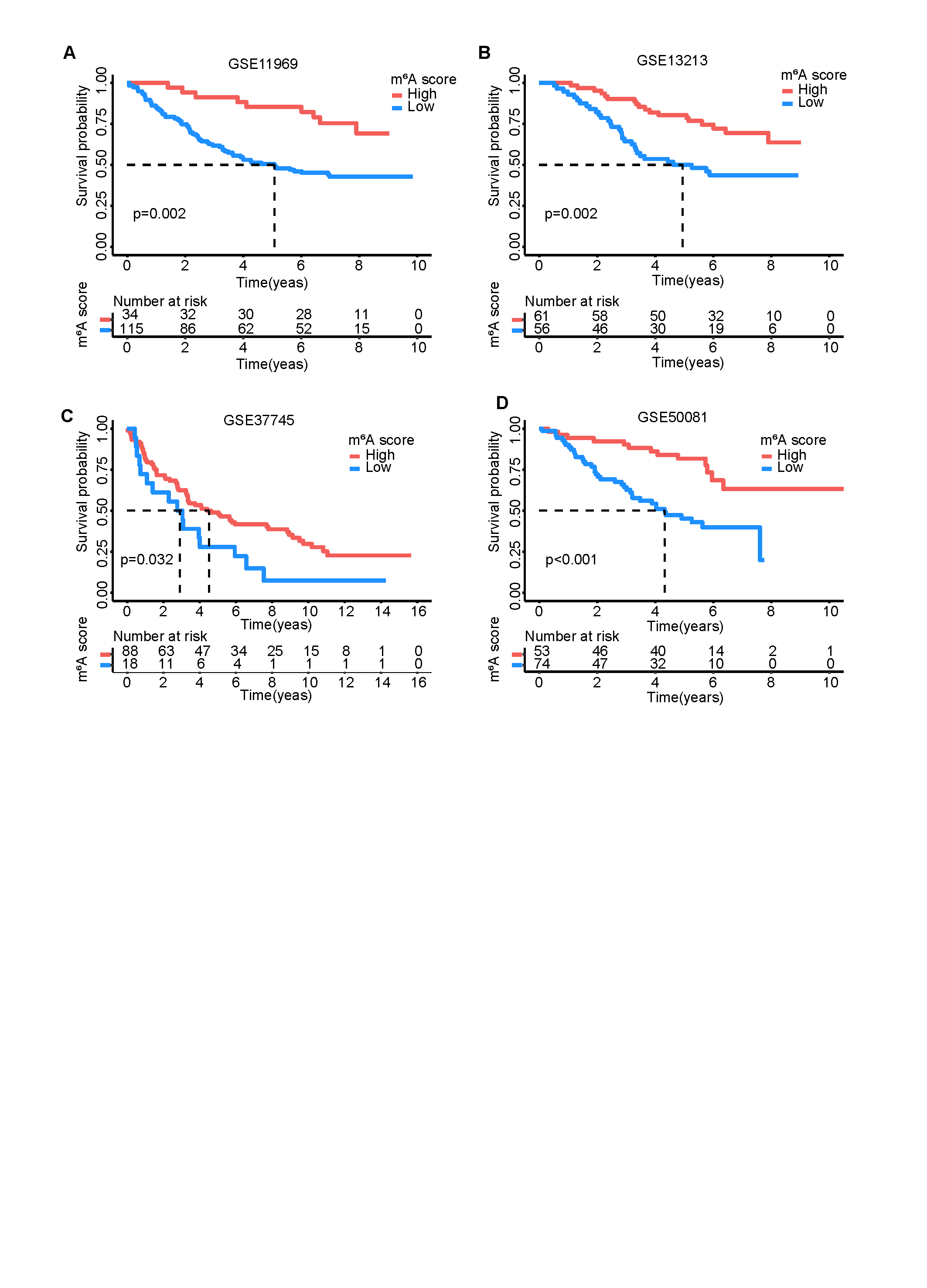

Supplement: Supplementary Figure S4 — External validation of the m6A score model in lung adenocarcinoma patients from GSE11969, GSE13213, GSE37745, and GSE50081 datasets. (A) Kaplan-Meier curves showing the differences in survival of the high and low m6A score groups in GSE11969 (P = 0.002, Log-rank test). (B) Kaplan-Meier curves showing the differences in survival of the high and low m6A score groups in GSE13213 (P = 0.002, Log-rank test). (C) Kaplan-Meier curves showing the differences in survival of the high and low m6A score groups in GSE37745 (P = 0.032, Log-rank test). (D) Kaplan-Meier curves showing the differences in survival of the high and low m6A score groups in GSE50081 (P < 0.001, Log-rank test). [file Image_4.tiff]
