# Supplementary material for: Characterization of m6A RNA Methylation Regulators Predicts Survival and Immunotherapy in Lung Adenocarcinoma
Source: Front Immunol. 2021 Dec 17;12:782551. doi: 10.3389/fimmu.2021.782551 (PMC8718692; doi:10.3389/fimmu.2021.782551)
Supplement: Supplementary file 5 [file Table_1.docx]

| Type | m^6^ARegulator | Name | Function |
| --- | --- | --- | --- |
| m^6^A writer | METTL3 | Methyltransferase-like 3 | Catalyzes m^6^A modification[12] |
|  | METTL14 | Methyltransferase-like 14 | Assists METTL3 to recognize the subtract[13] |
|  | METTL16 | Methyltransferase-like 16 | Catalyzes m^6^A modification[14] |
|  | WTAP | Wilms tumour 1-associated protein | Promotes METTL3-METTL14 heterodimer to the nuclear speckle[19] |
|  | KIAA1429 | vir-Like m^6^A methyltransferase associated | Guides the methyltransferase components to specific RNA region[20] |
|  | RBM15 | RNA binding motif protein 15 | Binds the m^6^A complex and recruit it to special RNA site[17] |
|  | RBM15B | RNA binding motif protein 15B | Binds the m^6^A complex and recruit it to special RNA site[18] |
|  | ZC3H13 | zinc finger CCCH-type containing 13 | Bridges WTAP to the mRNA-binding factor Nito[15] |
|  | CBLL1 | Cbl Proto-Oncogene Like 1 | Mediates mRNA splicing[17] |
|  | ELAVL1 | ELAV Like RNA Binding Protein 1 | Enhances mRNA stability[16] |
| m^6^A eraser | FTO | Fat mass and obesity-associated protein | Removes m^6^A modification[21] |
|  | ALKBH5 | Alkb homologue 5 | Removes m^6^A modification[22] |
| m^6^A reader | HNRNPA2B1 | Heterogeneous Nuclear Ribonucleoprotein A2/B1 | Promotes primary microRNA processing[31] |
|  | HNRNPC | Heterogeneous Nuclear Ribonucleoprotein C | Mediates mRNA splicing[30] |
|  | FMR1 | Fragile X mental retardation 1 | Enhances mRNA stability[29] |
|  | LRPPRC | Leucine Rich Pentatricopeptide Repeat Containing | mediates the export of nuclear mRNA[29] |
|  | YTHDF1 | YTH domain family 1 | Promotes mRNA translation[23] |
|  | YTHDF2 | YTH domain family 2 | Reduces mRNA stability[23] |
|  | YTHDF3 | YTH domain family 3 | Mediates the translation or degradation[24] |
|  | YTHDC1 | YTH domain containing 1 | Promotes RNA splicing and translocation[25] |
|  | YTHDC2 | YTH domain containing 2 | Enhances the translation of target RNA[26] |
|  | IGF2BP1 | IGF2 mRNA binding proteins 1 | Enhances mRNA stability[27] |
|  | IGF2BP2 | IGF2 mRNA binding proteins 2 | Enhances mRNA stability[28] |
|  | IGF2BP3 | IGF2 mRNA binding proteins 3 | Enhances mRNA stability[28] |

**Table S1** m^6^A RNA methylation regulators
